# Rectal Administration of Propylthiouracil in a Critically Ill Patient: A Life-Saving Experience

**DOI:** 10.7759/cureus.74817

**Published:** 2024-11-30

**Authors:** Yik Hin Chin, Siew Hui Foo

**Affiliations:** 1 Endocrinology and Diabetes, Hospital Selayang, Selayang, MYS

**Keywords:** acute abdomen, carbimazole, propylthiouracil (ptu), rectal administration, thyroid-storm

## Abstract

Hyperthyroidism is a common endocrine disease caused by the production of thyroid hormones in excessive amounts. Propylthiouracil (PTU) is one of the anti-thyroid drugs (ATD) used in the treatment of hyperthyroidism. Rectal PTU should be considered by physicians as a valuable option for managing hyperthyroidism as an alternative route of administration for patients who cannot tolerate oral medications. Our patient presented to the emergency department with acute abdomen. At the same time, he had thyroid storm which required the administration of oral PTU with other medications; however, post surgery, he was kept nil by mouth. Here, we highlight a case of the life-saving administration of rectal administration of propylthiouracil in a patient with a perforated gastric ulcer.

## Introduction

Hyperthyroidism is a common endocrine disease caused by production of thyroid hormones in excessive amount [1]. Propylthiouracil (PTU) is one of the anti-thyroid drugs (ATD) used in the treatment of hyperthyroidism. It belonged to a class of medications called thionamide antithyroid drugs. PTU works by inhibiting the production of thyroid hormones in the thyroid gland. It blocks the activity of an enzyme called thyroid peroxidase, which is involved in the synthesis of thyroid hormones (thyroxine (T_4_) and triiodothyronine (T_3_)). PTU also inhibits the iodization of tyrosine in the thyroid, thus reducing the synthesis of T_4_. This process allows PTU to disrupt the transformation of T_4 _to T_3_, which leads to a reduction in the level of serum Free T_3_ (FT_3_) [2]. PTU is usually given in oral preparation to hyperthyroid patients. In this report, we highlight a case of the life-saving administration of rectal administration of propylthiouracil in a patient with perforated gastric ulcer (PGU).

## Case presentation

A 35-year-old Burmese man presented to the emergency department (ED) with acute abdomen. He had severe abdominal pain for one day with a pain score of 8/10 and persistent vomiting for one day with poor oral intake. Also, he had no bowel output for two days prior to presentation. Abdominal palpation was noted to be tender with guarding.

The patient had been diagnosed with Graves’ disease almost six years prior to presentation and was managed in the medical outpatient clinic of Hospital Selayang. He was stable clinically and biochemically on carbimazole 10 mg once daily. He was seen at the surgical outpatient clinic for gastritis four months prior to the current presentation, where he was prescribed oral pantoprazole 40 mg once daily.

**Figure 1:**
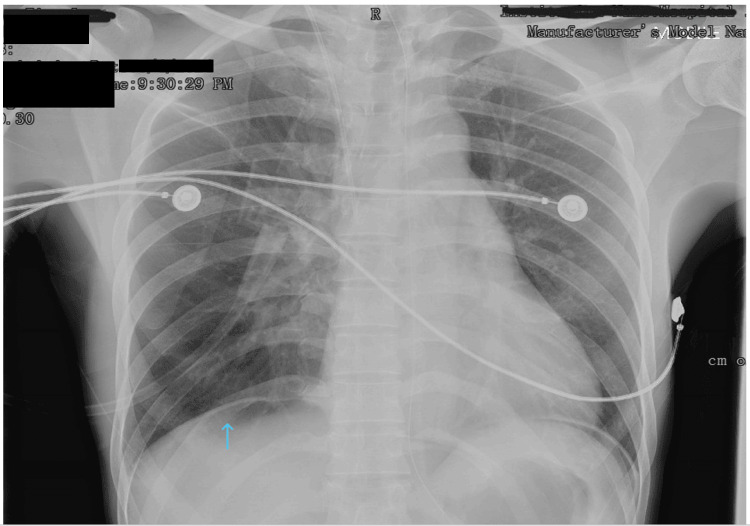
Abdominal X-ray showing air under diaphragm (blue arrow)

His condition was ill during the assessment by the ED team in the current presentation. Bedside abdominal scan showed free fluid over the Pouch of Morisson. Urgent erect chest radiograph (CXR) showed air under the diaphragm (Figure 1). Hence, an initial impression of perforated viscus was made. His blood pressure (BP) was noted to be low at 108/80 mmHg, heart rate of 146 beats per minute (bpm), and oxygen saturation of 100% under a nasal cannula. The patient was electively intubated for airway protection and impending circulatory shock.

During stabilization at the ED, an urgent thyroid function test (TFT) was sent. FT_4_ was 30.5 pmol/L while thyroid stimulating hormone (TSH) was 0.01 uIU/ml (Table 1). Bursch-Wartofsky score yielded 55 points which included heart rate: 25 points, agitation: 10 points, gastrointestinal symptoms: 10 points, and precipitating event: 10 points. The patient was treated for an impending thyroid storm and a loading dose of oral PTU 900 mg, Lugols iodine 10 drops, and hydrocortisone 100 mg intravenously were given in ED.

**Table 1:** Blood investigations taken during outpatient clinic check-up, on ED presentation (four months after clinic check-up), and three days after ED presentation and administration of rectal PTU 300 mg TDS. FT_4_: free thyroxine; TSH: thyroid stimulating hormone; WBC: white cell count; Hb: hemoglobin

Investigation/ reference values	Values on outpatient clinic check-up	Values on ED presentation	Values three days after intervention
FT_4 _pmol/L (7.86 -14.41	11.1	30	15.0
TSH uIU/ml (0.38 – 5.33)	0.01	<0.01	<0.01
WBC 10^9/L^ (4.0-10.0)		4.9	5.6
Hb g/dL (12.0 -15.0)		16.0	15.6
Platelet 10^9/L^ (150-410)		223	250
Sodium mmol/L (136-146)		131	130
Potassium mmol/L (3.4-4.5)		4.5	4.2
Creatinine umol/L (49-90)		124	115
Amylase U/L (25-200)		189	150

Subsequently, the patient underwent for exploratory laparotomy in the emergency operating room. Intraoperative findings include 1 cm perforation noted at the pre-pylorus region with a copious amount of peritoneal fluid at the right paracolic gutter, interloop of bowels, and pelvis region. Surgical mesh was employed during the repair and surgery was completed uneventfully. Postoperatively, he was transferred to the intensive care unit (ICU) for close monitoring.

He was kept nil by mouth for three days to allow the gastric perforation to seal. Thus, he was prescribed rectal administration of PTU 300 mg every eight hours in the ICU. PTU tablets were made into enema form by diluting with 90 ml of water for injection. Then, a Ryles tube was passed through the anal sphincter with a depth of about 10 cm. During the administration of rectal PTU, he was placed in the Trendelenburg position to minimize leakage of the PTU enema. This was given to him for three days.

The patient recovered well from the surgery with close monitoring in the ICU. He was extubated a day after the surgery with no complications. Repeated TFT in ICU showed a reduced FT_4_ of 15.0 mmol/L and TSH of 0.01 mmol/L. He was clinically euthyroid throughout the hospital stay. He was allowed orally on postoperative day 4 and rectal PTU enema was changed to oral carbimazole 20 mg once daily. Upon discharge, he was given oral carbimazole 10 mg once daily with a clinic visit in three months’ time.

## Discussion

Patients with thyrotoxicosis and severe intercurrent illness, or those undergoing a major surgical procedure, are at risk for developing thyroid storm. The mortality rate among such untreated patients approaches up to 75% [3]. In the current case, the patient presented with a life-threatening acute abdomen. Immediate measures included PTU loading dose, Lugol’s iodine, and hydrocortisone were administrated to reduce the risk of thyroid storm peri-operatively. However, after the surgery, he had to be kept nil by mouth by the surgeon for at least three days as the mesh was in place to allow the perforation to seal.

PTU has been commonly used in the acute management of thyroid storm [4]. PTU can be administered regularly every four to eight hours. PTU is favored over methimazole because of its effect in inhibiting T_4_ to T_3_ conversion. T_3 _levels drop by approximately 45% within 24 hours after PTU administration as compared to only 10-15% within 24 hours after methimazole administration [2]. Therefore, in the case of a thyroid storm, PTU is preferred for its rapid onset and efficacy in lowering T_3_ levels.

The rectal route of drug administration remains a practical alternative to clinicians as drugs can be administered for both local and systemic actions. It is a widely acceptable choice in circumstances where the oral route is not feasible. The environment in the rectum is considered relatively constant and stable. As compared to the gastrointestinal tract, the rectum has low enzymatic activity causing less interaction with drugs. In addition, drugs can bypass the liver, which reduces the hepatic first-pass effect. Therefore, the rectal route of drug administration can provide significant local and systemic levels to the body [5].

Rectal administration of PTU has been studied and suggested in many clinical practice guidelines. Intravenous form of thioamides is not readily available [6]. PTU usage in clinical practice has been observed as early as 1988 [7]. It is not commonly used in daily clinical practice but in situations where the oral route is not feasible, PTU can be absorbed effectively via the rectal route in the form of a suppository or enema. The serum levels of PTU given via this route were shown to be within the high therapeutic range for up to five days [8]. 

Both forms have been proven to be able to significantly decrease serum FT_3_ levels shortly after administration with comparable therapeutic effects. However, the enema form appeared to provide better bioavailability than the suppository form [9]. This nursing team in the ICU has been trained to prepare PTU in both forms. Administration of PTU via rectal enema was simple and yet effective. The patient in this case responded well to the rectal enema as repeated FT_4 _showed a significant reduction from 30.0 to 15.0 pmol/L. Upon clinical improvement, the preparation can be easily converted back to oral administration, as was done in this case.

## Conclusions

Rectal PTU should be considered by physicians as a valuable option for managing hyperthyroidism as an alternative route of administration for patients who cannot tolerate oral medications. Even though it is not commonly used, this case serves as a good example of rectal PTU efficacy. However, its use should be carefully considered in the context of the individual’s needs and preferences, along with other available treatment options.

## References

[1] Ross DS, Burch HB, Cooper DS (2016). 2016 American Thyroid Association guidelines for diagnosis and management of hyperthyroidism and other causes of thyrotoxicosis. Thyroid.

[2] Cooper DS, Saxe VC, Meskell M, Maloof F, Ridgway EC (1982). Acute effects of propylthiouracil (PTU) on thyroidal iodide organification and peripheral iodothyronine deiodination: correlation with serum PTU levels measured by radioimmunoassay. J Clin Endocrinol Metab.

[3] Walter RM Jr, Bartle WR (1990). Rectal administration of propylthiouracil in the treatment of Graves' disease. Am J Med.

[4] Lee SY, Modzelewski KL, Law AC, Walkey AJ, Pearce EN, Bosch NA (2023). Comparison of propylthiouracil vs methimazole for thyroid storm in critically ill patients. JAMA Netw Open.

[5] Accomasso L, Cristallini C, Giachino C (2018). Risk assessment and risk minimization in nanomedicine: a need for predictive, alternative, and 3Rs strategies. Front Pharmacol.

[6] Wiggins A, Leschorn H, Tekwani S (2021). An alternative route of treatment for refractory vasodilatory shock due to thyroid storm. Chest.

[7] Bartle WR, Walker SE, Silverberg JD (1988). Rectal absorption of propylthiouracil. Int J Clin Pharmacol Ther Toxicol.

[8] Zweig SB, Schlosser JR, Thomas SA, Levy CJ, Fleckman AM (2006). Rectal administration of propylthiouracil in suppository form in patients with thyrotoxicosis and critical illness: case report and review of literature. Endocr Pract.

[9] Jongjaroenprasert W, Akarawut W, Chantasart D, Chailurkit L, Rajatanavin R (2002). Rectal administration of propylthiouracil in hyperthyroid patients: comparison of suspension enema and suppository form. Thyroid.

